# Ovariotomy

**Published:** 1850-04

**Authors:** 


*Ovariotomy.*—We have received the following note from Dr. W. L. Atlee on this subject:

Dear Doctor: Since I placed in your hands the report of my fourth and fifth cases of large peritoneal section, I have performed the operation of gastrotomy twice. Both patients have recovered from the operation, and no unfavorable symptom interfered with their recovery.

The sixth operation, which was exploratory, was on the 13th of October, the patient, Miss M. B., aged 43 years; the incision extending from one inch above the umbilicus to the pubis; the tumour, uterine, and not adherent. After deciding against its extirpation, the wound was closed.

The seventh operation was on the 24th of November: the patient, Mrs. T. H., aged 39 years; the incision of the same length, the tumour fibrous, attached by a very thick dense pedicle to the fundus uteri, weighing six pounds.

Chloroform was employed in both cases in the same way as in the previous operations, and with the same happy results.

A report of these cases will be prepared for the "*American Journal of the Medical Sciences.*" Very respectfully, &c.,

Washington L. Atlee.

To Isaac Hays, M. D.

*Philadelphia*, Dec. 22d, 1849. *Medical News.*

